# On the Kind of Union Obtained after Fractures

**Published:** 1845-09

**Authors:** 


### ON THE KIND OF UNION OBTAINED AFTER FRACTURES.

A late number of the *Buffalo Medical Journal*, in "Notes of an European Tour," by Prof. Hamilton, contains a table exhibiting the condition of the specimens of fractural bones preserved in the *Musée Dupuytren*, at Paris. By this it would appear that out of 180 fractures, no union occurred in 25, imperfect union in 57, crooked and shortened 43, nearly or quite perfect 55. From these results the conclusion is drawn that "most surgeons are in error in regard to the power they possess in these cases." It is highly desirable that proper attention should be drawn to this subject, and reliable information in regard to it disseminated; although a mere collection of broken bones without any means of ascertaining either the kind of fracture, the treatment employed, or the circumstances which affect it favorably or otherwise, can do but little toward ascertaining the effect of judicious treatment in any given variety of fracture. Were those fractures from fire-arms? Did they occur in the military service? Had they any surgical treatment? These are questions which, in reference to many of them, cannot be answered.

It will be observed, that in the subjoined table certain fractures, as of the clavicle, and neck of the femur within the capsule, appear to result quite as favorable as could well be anticipated in private practice; while of others, as of the humerus, the result is much less satisfactory.

It is obvious, that in compiling statistics of this sort, results widely different will be arrived at by examining dried specimens, and those of the same cases, while the subjects were living. Many which, situated deeply beneath the muscles, give no evidence of deformity and are highly useful during life, are found, after death, to be more or less imperfect.

For a vicious direction, or even a shortening of the limb, if but slight, does not necessarily produce lameness, or interfere in any way with the movements of the member. For this reason we do not add to the statistics of the *Musée Dupuytren* the results of the cases that have, in numerous large hospitals, in private practice of others as ourselves, fallen under our observation as being only seen during life; and the number of specimens of fractured bones of which we could furnish descriptions, would be inconsiderable when compared with those deposited in the various museums. We may add, that from the considerations given, we do not see reason to doubt that the received opinions in regard to the effect of the treatment of fractures, all circumstances being favorable, are correct, and that in such cases a member nearly perfect may be generally procured. The only point in which we have been led to form a different opinion is in reference to those involving the articulations, in which the obstacles to a restoration of the movements of the member are, in numerous cases, insuperable.

| Names of Bones. | Total Number. | No union | Union Fibro cellular | Union Fibro - cellular and ossific. | Union by bone, but crooked. | Union by bone, but shorten'd | Both crooked & short. | Union nearly or quite perfect. |
|---|---|---|---|---|---|---|---|---|
| Inferior Maxilla, | 2 | | | | 1a | | | 1 |
| Clavicle, | 12 | | | | 8 | | | 4 |
| Scapula, | 3 | | | | 1 | | | 2 |
| Humerus, | 21 | 2 | | | 8 | 2 | 6 | 3 |
| Radius and Ulna, | 2 | | | | | | | 2 |
| Shaft of Ulna, | 7 | 1 | | | 2 | | | 4 |
| Olecranon. | 2 | | | | 1b | | | 1 |
| Shaft of Femur, | 49 | 2 | | | | | 37c | 10 |
| Neck of F. extra capsul. | 12 | 2 | 1 | 2 | 1 | | | 6 |
| Neck of F. intra capsular | 24 | 16d | 5 | | | | | 3 |
| Patella, | 8 | | 7e | | | | | 1 |
| Tibia, | 13 | 1f | | | 7 | | | 5 |
| Fibula, | 11 | 1 | | | 3 | | | 7 |
| Tibia and Fibula, | 12 | | | | 4 | 2 | | 6 |
| | 178 | 25 | 13 | 2 | 36 | 4 | 43 | 55 |

a. Left condyle displaced inward.
b. United by bone, but fragment much displaced.
c. One united at nearly a right angle with the proper direction; 5 are shortened two inches and a half; one, a transverse fracture, is shortened three inches and a quarter.
d. Of these, eight are recent fractures, and eight are ancient, having formed false joints.
e. No. 205, is separated 4 inches, and No 206, six inches.
f. False joint.

